# Post-harvest processing methods have critical roles on the contents of active metabolites and pharmacological effects of *Astragali Radix*

**DOI:** 10.3389/fphar.2024.1489777

**Published:** 2024-12-09

**Authors:** Xiaoyan Zhang, Shengnan Jiang, Tingting Sun, Wenbing Zhi, Kairu Ding, Ziyao Qiao, Hong Zhang, Ye Li, Yang Liu

**Affiliations:** ^1^ Institute of Chinese Medicine, Shaanxi Academy of Traditional Chinese Medicine (Shaanxi Traditional Chinese Medicine Hospital), Xi’an, China; ^2^ College of Life Sciences, Northwest University, Xi’an, China

**Keywords:** *Astragali Radix*, fresh processing, traditional processing, active metabolites, pharmacological effects

## Abstract

**Background:**

Processing methods of traditional Chinese medicinal materials are critical in influencing the active metabolites and pharmacological effects. The fresh processing method effectively prevents the loss and degradation of metabolites, common in traditional drying and softening processes, while also reducing production costs. *Astragali Radix* (AR), a leguminous botanical drug, is widely utilized in clinical practice and functional foods. Therefore, optimizing its the post-harvest processing method is crucial for enhancing production and application.

**Methods:**

AR samples were processed using different methods with varying moisture content, including unpretreated samples and those subjected to kneading and sweating treatments. These samples were evaluated for physical appearance and active metabolite content. The entropy weight method, combined with the technique for order preference by similarity to ideal solution (TOPSIS), was employed to optimize the fresh processing method. A comparative study between freshly processed AR (AR-F) and traditionally processed AR (AR-T) assessed active metabolites, pharmacological effects and mechanisms.

**Results:**

The appearance and active metabolites content of AR samples were affected by moisture content, kneading, and sweating treatments. After these treatments, the content of polysaccharides and calycosin-7-O-glucoside increased compared to unpretreated samples at the same moisture level. Entropy weight-TOPSIS analysis showed that the sample with 35% moisture, 100 kneading cycles, and 12 h of sweating had the highest score. Comparative studies revealed that AR-F had significantly higher content of total polysaccharides, total flavonoids, and calycosin-7-O-glucoside compared to AR-T, along with an increased flavonoid glycoside/aglycone ratio. However, no significant differences were observed in the total saponins and their metabolites. Pharmacological studies demonstrated that total flavonoids in AR-F exhibited superior macrophage RAW264.7 activation, compared to AR-T. Furthermore, we confirmed that the enhanced immunomodulatory capacity of AR-F was linked to its ability to stimulate the release of TNF, SRC, ER-α, AKT, HSP90, and Caspase-3 in RAW264.7 cells.

**Conclusion:**

Our study optimized the fresh processing method of AR, and conducted a systematic comparative analysis between fresh and traditional processing samples, providing a basis for post-harvest processing in the AR production areas.

## 1 Introduction

The processing of traditional Chinese medicinal materials is a crucial aspect of Traditional Chinese Medicine (TCM) theory, significantly influencing their chemical composition and pharmacological activity. The fresh processing method involves cleaning freshly harvested medicinal materials, cutting them into slices, segments, blocks, or sections, and then drying them. This approach aligns with the concept of fresh processing recorded in “Leigong’s Pharmacopoeia” from the Northern and Southern Dynasties (Yang et al., 2016). In contrast to traditional processing methods, which require drying and soaking before cutting, fresh processing methods eliminate these steps. This effectively saves time and prevents the loss of volatile, water-soluble, enzyme-active, and heat-sensitive metabolites. Additionally, this method reduces the risk of quality non-conformance and lowers production costs. Studies have shown that fresh-cut processing results in different chemical profiles compared to traditional methods. For example, the total saponin and ginsenoside content in freeze-dried *Panax notoginseng* slices processed using fresh-cut methods increased by 16.5% and 22.5%, respectively, compared to traditional methods (Liu et al., 2019). Similarly, the contents of cinnamaldehyde, cinnamic acid, essential oil, and extractives in fresh-cut cinnamon slices were higher than those in traditionally processed slices (Luo et al., 2017). Differences in effective metabolite content can lead to changes in pharmacological activity. For instance, fresh-cut *Cortex phellodendri* slices demonstrated enhanced antipyretic effects in heat syndrome rats and greater anti-inflammatory effects in acute bacterial peritonitis rats compared to traditional slices. Additionally, at the same dosage, fresh-cut *Sophora* flavescens slices showed a significantly higher inhibition rate of ear swelling in mice induced by xylene compared to traditional slices (Chen et al., 2022a). These studies suggest that fresh processing may enhance efficacy through better metabolite retention, potentially reducing the required dosage to achieve the same therapeutic effects, thereby minimizing side effects and safety risks, which is of significant clinical importance.

*Astragali Radix* (AR), a leguminous botanical drug, encompasses both *Astragalus membranaceus* (Fisch) Bge. var. mongholicus (Bge.) Hsiao and *Astragalus membranaceus* (Fisch) Bge (China, 2020). The roots of this plant are revered as a supreme tonic for Qi and are widely used in TCM formulations and preparations. Known for their efficacy in treating tumors, enhancing immunity, and addressing pulmonary diseases, they are also commonly included in dietary supplements due to their anti-aging, antioxidant, hypoglycemic, and antihypertensive effects (Zhang et al., 2021). Research has identified polysaccharides, saponins, and flavonoids as the three primary active metabolites contributing to AR’s pharmacological effects (Liu et al., 2024). Among these, polysaccharides are present in significantly higher amounts, ranging from 5% to 20%, compared to saponins (0.5%–3%) and flavonoids (0.1%–1%) (Li et al., 2022; Li et al., 2020). Notably, the Chinese Pharmacopoeia only includes the saponin Astragaloside IV and the flavonoid calycosin-7-O-glucoside as quality control markers for AR. The traditional processing steps for AR slices involve removing impurities, washing, drying, moistening, slicing, and drying again, which adds complexity to the process. During the “moistening” phase, polysaccharides and other glycosides, due to their high water solubility, may lose metabolites, potentially affecting their efficacy.

Therefore, optimizing the moisture content during fresh processing is crucial for preserving active metabolites, ensuring quality, and improving processing efficiency (Zhang et al., 2023; Yao et al., 2024). Kneading and sweating are traditional processing methods used in TCM, which has been observed that fresh roots subjected to these repeated compressions and heating conditions become more flexible and smooth, reducing microbial growth and facilitating slicing and storage. This process also fosters biochemical and chemical transformations within the material, enhancing its efficacy (Wu et al., 2021). Modern processing trends emphasize integrating processing at the production site, allowing AR to be directly sliced without the cumbersome steps of drying and moistening, resulting in more stable and standardized quality. However, the complexity of the material, the diversity of evaluation criteria, and differences in analytical methods often complicate efforts to establish a universally recognized “optimal” processing method.

Our study aims to systematically investigate the fresh processing of AR, focusing on the impact of moisture content, kneading, and sweating on quality. Indicators such as physical appearance, extractives, total polysaccharides, Astragaloside IV, and calycosin-7-O-glucoside have been evaluated. The entropy weight method combined with TOPSIS analysis was employed to determine the optimal processing conditions. Additionally, a comparative study was conducted on fresh and traditional processing AR in terms of total polysaccharides, total saponins, total flavonoids, and the content of four saponin and eight flavonoid metabolites. Furthermore, we focused on the flavonoid content differences between AR-T and AR-F, and clarified the immune-regulatory activity and mechanism of AR-F and AR-T through network pharmacology, molecular docking and *in vitro* cell experiments. This research provided a basis for the post-harvest processing in the producing area of AR.

## 2 Materials and methods

### 2.1 Materials

The fresh roots of AR were harvested from Guangji Hall Pharmaceutical Group Co., Ltd. In Shaanxi Province and identified by researcher Hong Zhong from the Shaanxi Academy of Traditional Chinese Medicine. Standard reference materials, including Astragaloside IV, I, II, III, calycosin-7-O-glucoside, calycosin, mangiferin, mangiferin aglycone, and astragaloside A, B, C, and D, were purchased from Manster Technology Co., Ltd. (Chengdu, China), with purities exceeding 98%. The ABTS total antioxidant capacity assay kit (A015-2-1) was obtained from Nanjing Jiancheng Bioengineering Institute. 1,1-Diphenyl-2-picrylhydrazyl (DPPH, D861666) and p-nitrophenyl-α-D-glucopyranoside (PNPG, N814753) were purchased from Macklin (Shanghai, China), while α-glucosidase (S10050) was obtained from Shanghai Yuan Ye Bio-Technology Co., Ltd. Lipopolysaccharide (LPS, L2880) was acquired from Solarbio (Beijing, China). Nitric oxide (NO) assay kits were provided by Biyuntian Co., Ltd. (Shanghai, China). Chromatographic grade acetonitrile and formic acid were purchased from Thermo Fisher Scientific (United States). All other chemicals and reagents used were of analytical grade. TNF, SRC, ESR1, EGFR, AKT1, HSP90AA1 and CASP3 ELISA kit were obtained from FineTest (Wuhan, China).

### 2.2 Optimization of fresh processing method of AR

#### 2.2.1 Sample preparation

Fresh Astragalus roots, free from damage, rot, or black spots, and of uniform size (approximately 1.0–1.2 cm in diameter and 3.5 cm below the top cut of the root) were selected. The roots were cleaned to remove soil and root hairs, then divided into 12 groups, each weighing 5 kg and consisting of three replicates. Six groups were dried at 40°C to achieve different moisture levels (X1: 55%; X2: 45%; X3: 40%; X4: 35%; X5: 25%; X6: 15%), then cut into 4 mm thick slices and further dried at 40°C until the moisture content fell below 10.0%. The other six groups were similarly dried at 40°C to different moisture levels (Y1: 55%; Y2: 45%; Y3: 40%; Y4: 35%; Y5: 25%; Y6: 15%), then kneaded 100 times, sealed, and allowed to rest for 12 h. The samples were then cut into 4 mm thick slices and dried again at 40°C until the moisture content was below 10.0%. All procedures were repeated three times to ensure consistency.

#### 2.2.2 Appearance and scoring

The appearance of the samples was evaluated according to the Chinese Pharmacopoeia (2020). To comprehensively assess the quality of the samples, factors such as texture, slicing continuity, color, proportion of curled slices, and proportion of fragments were considered, with scoring criteria outlined in Table 1.

**TABLE 1:** Scoring criteria for the appearance of fresh-processed AR.

Evaluation indicator	Criteria	Score
Texture	Very brittle (difficult to slice)	0
	Slightly soft (easier to slice)	0.5
	Bendable without breaking (easy to slice)	1
Slicing continuity	Many continuous slices	0
	Few continuous slices	0.5
	No continuous slices	1
Color	Cut surface: pale white; Wood: pale white	0
	Cut surface: pale yellow-white; Wood: pale white	0.5
	Cut surface: yellow-white; Wood: light yellow	1
Proportion of curled slices	Many curled slices (≥20%)	0
	Few curled slices (10%–20%)	0.5
	Very few curled slices (≤10%)	1
Proportion of fragments	Many fragments (≥20%)	0
	Few fragments (10%–20%)	0.5
	Very few fragments (≤10%)	1

#### 2.2.3 Determination of moisture, total ash, and water-soluble extract

The moisture, total ash and water-soluble extract content were assessed using methods 08323, 2,302, and 2,201 respectively, from the Chinese Pharmacopeia 2020 edition (China, 2020).

#### 2.2.4 Determination of total polysaccharides, astragaloside IV, and isoflavone glycoside

Total polysaccharides were measured using the phenol-sulfuric acid method (Jiang et al., 2016). Astragaloside IV and Isoflavone glycoside were quantified according to the methods from the Chinese Pharmacopoeia 2020 edition (China, 2020).

#### 2.2.5 Entropy Weight-TOPSIS analysis

The entropy weight method is an objective weighting approach that constructs a judgment matrix for each evaluation index to derive the entropy of each index. The entropy weight is determined based on these entropy values; lower information entropy indicates a higher degree of dispersion for the index and, consequently, a higher weight. The TOPSIS method addresses multi-criteria evaluation and ranking problems by first calculating the index weights using the entropy weight method, and then identifying the best and worst solution vectors. The Euclidean distance measures the distance between each evaluation index and the positive ideal solution (D^+^) or negative ideal solution (D^−^). Comprehensive scores (C_*i*_) are calculated based on these distances, ranking the samples to assess the quality of each AR fresh-cut processing method (Xu et al., 2022; Zhou et al., 2017; Wang et al., 2019; Shin et al., 2007).

### 2.3 Comparison of active metabolites content between AR-F and AR-T

#### 2.3.1 Sample preparation

Fresh Astragalus roots, free from damage, rot, or black spots, and of uniform size, were selected and cleaned. The roots were divided into two groups, each weighing 10 kg and consisting of three replicates. For the fresh-cut method, the processing involved drying the fresh AR roots at 40°C until the moisture content reached 35%, followed by kneading 100 times, sealing, and piling for 12 h, and then slicing to produce AR-F. AR-T was processed adhered to the preparation guidelines outlined in the 2020 edition of the Chinese Pharmacopoeia for Astragalus, which included specific traditional methods such as steaming under atmospheric pressure for 5 min, as determined through preliminary trials.

#### 2.3.2 Determination of total polysaccharides, total saponins, and total flavonoids

Total polysaccharides were measured using the phenol-sulfuric acid method (Jiang et al., 2016), while total saponins were assessed using the vanillin-ice acetic acid-perchloric acid method (Li et al., 2021). Specifically, 0.5 g of AR powder was mixed with 25 mL methanol and refluxed for 1 h. After filtering, methanol was recovered under reduced pressure. The residue was subsequently extracted with 25 mL water-saturated butanol in three portions, which was then reduced to dryness. The resulting residue was dissolved in methanol, and a portion was derivatized with vanillin-ice acetic acid and perchloric acid before measuring absorbance at 539 nm. Astragaloside IV served as the reference for calculating total saponin content. Total flavonoids were measured using the method outlined in the Chinese Pharmacopoeia (2020 edition). Sample preparation involved extraction with ethanol, followed by measurement at 280 nm using HPLC-DAD.

#### 2.3.3 Monosaccharide composition analysis

Monosaccharide composition was analyzed through acid hydrolysis, acetylation, and GC methods. Initially, 15 g of AR powder was extracted with 300 mL of water at 90°C for 4 h, after which the supernatant was concentrated. Proteins were subsequently removed using enzyme and trichloroacetic acid methods, resulting in crude polysaccharides that were obtained by precipitation with ethanol. Each polysaccharide samples (5 mg) underwent hydrolysis with trifluoroacetic acid, followed by acetylation and reduction. Mixed standard samples of fucose, rhamnose, arabinose, xylose, mannose, glucose, galactose, glucuronic acid, and galacturonic acid were prepared similarly. The analysis was performed using GC under the following conditions: Rtx-50 column, a temperature gradient from 180°C to 240°C, with detection via FID.

#### 2.3.4 Determination of saponin content

4.0 g of AR powder was extracted with 40 mL of methanol through two refluxing sessions. The filtrate was then concentrated and dissolved in water, followed by extraction with water-saturated butanol. After concentrating the butanol layer, the residue was dissolved in methanol. Saponin content was subsequently analyzed using HPLC-ELSD, utilizing an Agilent ZORBAX SB-Aq column with gradient elution using acetonitrile and water at specific detector temperatures.

#### 2.3.5 Determination of flavonoid content

Flavonoid content was quantified using HPLC-DAD, following the sample preparation guidelines from the Chinese Pharmacopoeia (2020 edition) for isoflavone glycosides. The analysis was conducted on an Agilent ZORBAX SB-Aq column with gradient elution, measuring detection at 230 nm and 260 nm.

### 2.4 Pharmacological effect mechanism of AR-F and AR-T

#### 2.4.1 Targets prediction of differential metabolites

Based on a previous study, we obtained the differentiated metabolites of AR-F and AR-T, and screened the potential metabolites using the Swiss ADME database. The metabolites were selected by applying the condition of at least two “yes” responses for Druglikeness. The 3D structures of the metabolites that met this criterion were then imported into the SwissTargetPrediction database, where potential targets were identified with a probability greater than 0.1.

#### 2.4.2 Construction of the PPI network and identification of core targets

The potential targets were imported into the STRING database with the species set to human and the confidence level set to the highest. The protein interaction network was obtained by excluding isolated proteins, and the data were imported into Cytoscape 3.7.2 to construct the PPI network. Topological analysis was performed based on node degree, closeness, and betweenness. Targets with lower degrees were excluded, and the core targets were identified using the CentiScape plugin, selecting those with degree, closeness, and betweenness values greater than the median.

#### 2.4.3 Enrichment analyses using GO and reactome databases

Core targets were subjected to GO and Reactome enrichment analyses using the DAVID. The enrichment results were then visualized through an online bioinformatics mapping platform and presented as a bubble chart.

#### 2.4.4 Molecular docking analysis

The human protein structure files of the top seven core targets in the PPI network were downloaded from the PDB database. The 3D structures of the target-interacting metabolites were retrieved from the PubChem database. The proteins were pre-treated by dehydration and hydrogenation using PyMol. Molecular docking between the ligand molecules and the proteins was calculated using AutoDock 1.5.7 software.

#### 2.4.5 Extraction and purification of total flavonoids of AR (TFA)

Briefly, the dried slices of AR-F and AR-T were macerated in 95% ethanol and then extracted. The ethanol extraction was repeated 3–4 times and the pooled extract then was concentrated to a specific gravity of 1.35 by evaporation. The concentrated solution was extracted with ethyl acetate. The concentrated ethyl acetate extract was further separated by silica gel columns. The main chemical components of the purity of TFA exceeded 90%.

#### 2.4.6 Immunomodulatory effects

##### 2.4.6.1 Cell culture and viability assay

RAW264.7 mouse macrophage cells were purchased from ACTT Chinese Cell Resource Bank and cultured in high-glucose DMEM with 10% fetal bovine serum at 37°C with 5% CO_2_. Cell viability was measured using the MTT assay. The cells were seeded in a 96-well plate at a density of 1 × 10^4^ cells/well, and the supernatant was discarded after the cells adhered to the wall; 100 μL of DMEM culture medium containing different crude drug concentrations (32.5∼1,000 μg/mL) of TFA was added, with DMEM culture medium as the blank control, and 6 replicate wells were set for each concentration. After incubation for 24 h, MTT (5 mg/mL) was added and incubated for 4 h, DMSO was added to dissolve the formazan crystals in the cells, and the absorbance was measured at 490 nm.

##### 2.4.6.2 NO content measurement

The cells were seeded in a 96-well plate at a density of 1 × 10^4^ cells/well. After cell adhesion, the supernatant was discarded, and 100 μL of DMEM culture medium containing different concentrations (32.5–125 μg/mL) of TFA was added, with DMEM culture medium as the blank control and LPS (1 μg/mL) as a positive control. Six replicate wells were established for each concentration. After 24 h of incubation, the cell supernatants were collected, and NO release was measured using the Griess method.

#### 2.4.7 Enzyme-linked immunosorbent assay

Cells in the logarithmic growth phase were cultured. The blank control group received DMEM high glucose complete medium, the drug administration groups were treated with TFA at concentrations of 31.25, 62.5 and 125 μg/mL, and the positive control group received 10 μg/mL LPS solution. After 24 h of incubation, cell supernatants were collected, and ELISA was performed to measure TNF, SRC, and ESR1 expression. Cells were inoculated into 12-well plates, and after culturing, proteins were extracted via repeated freeze-thawing in liquid nitrogen. AKT1, CASP3, and HSP90AA1 expression in cells was also detected by ELISA (Saddiq et al., 2022).

### 2.5 Statistical analysis

Data are presented as mean ± standard deviation. For analytical and cell experiments, averages were based on three and six independent replicates, respectively. Statistical analysis was performed using GraphPad Prism 10 (San Diego, CA, United States).

## 3 Results

### 3.1 Optimization of fresh processing method of AR

#### 3.1.1 Appearance of samples

**FIGURE 1:**
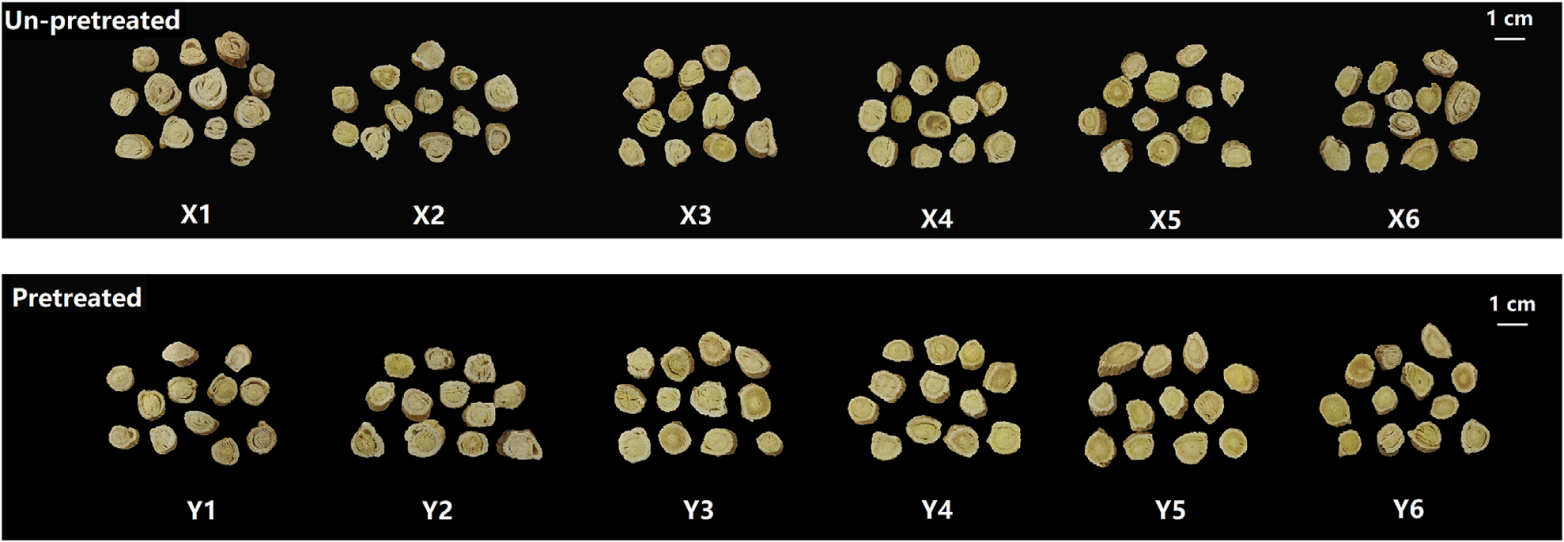
Appearance characteristics of AR with different fresh-processed methods.

**FIGURE 2:**
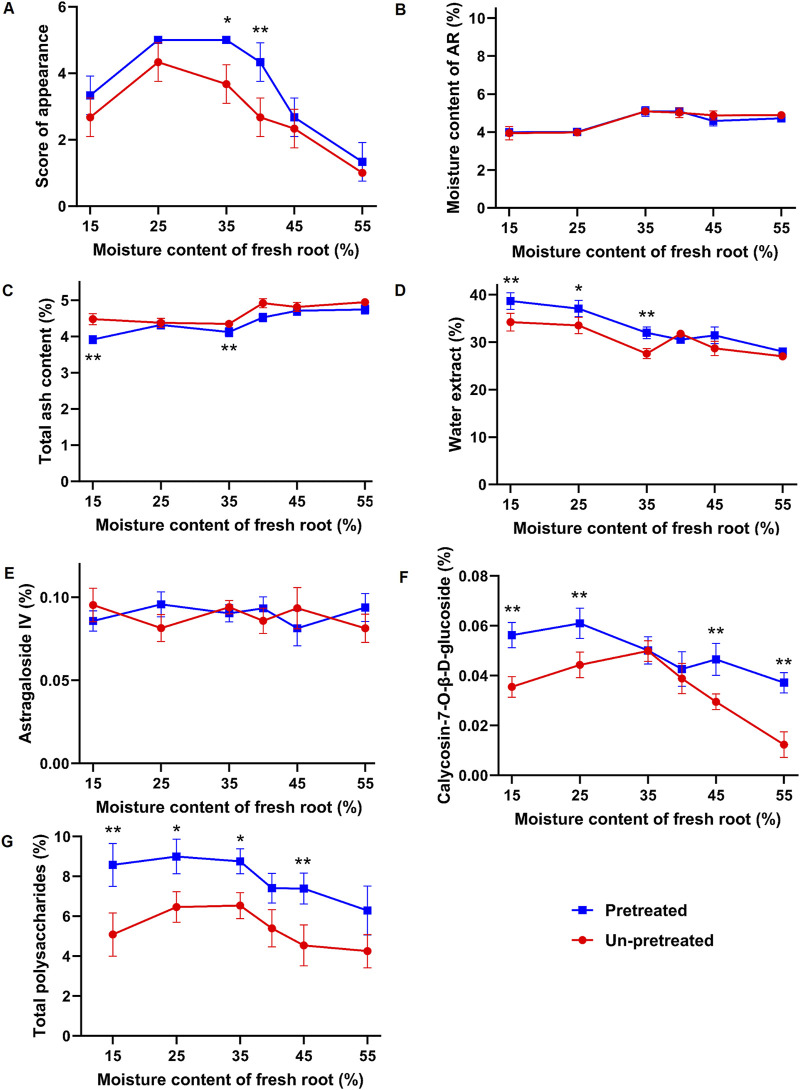
Effects of different moisture contents and processing methods on the appearance and intrinsic properties of fresh-processed AR. **(A)** Appearance score. **(B)** Moisture. **(C)** Total ash. **(D)** Water-soluble extract. **(E)** Astragaloside IV. **(F)** Calycosin-7-O-β-D-glucopyranoside. **(G)** Total polysaccharides. Compared with the un-pretreated group with the same moisture content, ^*^ *p* < 0.05, ^**^ *p* < 0.01.

The appearance of samples prepared using different methods is shown in Figure 1. When the moisture content of the material was between 45% and 55%, samples X1-X3 and Y1-Y3 often exhibited curled and peeling epidermis, separation of the bark and wood parts, and higher rates of warping and breakage. As the moisture content decreases to between 35% and 25%, these issues improved, resulting in fewer instances of warping and breakage. However, when the moisture content dropped further to 15%, samples X6 and Y6 became hardened and brittle at the ends, leading to fragmentation and affecting the yield of the medicinal slices. The kneading and sweating pretreatment had a noticeable impact on the appearance of the samples. Samples Y1-Y6, which were kneaded and sweated before cutting, showed significant improvements compared to samples X1-X6 which were cut directly. The pretreatment reduced bark-wood separation, resulting in more compact and dense slices with fewer instances of warping and breakage. Color is also a significant quality indicator for medicinal materials. According to pharmacopoeia standards, the cut surface of samples should exhibit a distinct color contrast between the yellowish wood part and the white bark, forming the characteristic “golden well and jade rail”. As shown in Figure 1, samples X1-X3 and Y1-Y3 had a lighter wood color, resulting in a less pronounced contrast with the bark. In contrast, samples X4-X6 and Y4-Y6 exhibited increasingly darker wood colors, better displaying the “golden well and jade rail” feature. Based on the scoring criteria in Table 1, the appearance of the 12 fresh-cut processing methods was evaluated (Figure 2A). Samples Y4 and Y5 received the highest appearance scores of 5 points. Across all moisture levels, the appearance scores of the pretreated group were consistently higher than those of the unpretreated group.

#### 3.1.2 Content of moisture, total ash, and water-soluble extract in samples

The moisture, total ash, and water-soluble extract content of samples are shown in Figure 2B-D. All groups met the pharmacopoeia standard for moisture content (not exceeding 10.0%). However, Sample X1 had the highest total ash content, exceeding the pharmacopoeia limit of 5.0%, followed by X3, which was also significantly higher than Y4-Y6. Regarding water-soluble extract content, all groups met the pharmacopoeia standard (not less than 17.0%). Samples Y5 and Y6 had the highest water-soluble extract content, measuring 37.03% ± 1.72% and 38.67% ± 1.77%, respectively, which were significantly higher than those of the other fresh-cut slices.

#### 3.1.3 Active metabolites content of samples

The content of active metabolites in samples with different moisture levels showed varying trends. The content of Astragaloside IV (Figure 2E) fluctuated within the moisture range of 15%–55%. Except for sample X1, which had a content of 0.0813 ± 0.0085 (below the pharmacopoeia standard of not less than 0.080%), the contents in other samples were within the standard, with sample Y4 having the highest content of 0.103 ± 0.0076, with no significant differences among the samples. The content of calycosin-7-O-β-D-glucopyranoside (Figure 2F) displayed a trend of increasing and then decreasing with varying moisture content. It increased from 15% to 35% moisture content and decreased from 35% to 55% moisture content. Sample X1 had the lowest content of 0.0123 ± 0.0051, which did not meet the pharmacopoeia standard (not less than 0.020%), while sample Y4 had the highest content of 0.0388 ± 0.0061.

In samples subjected to kneading and sweating treatment, the content of calycosin-7-O-β-D-glucopyranoside increased with decreasing moisture content from 55% to 25%, reaching a maximum in sample Y5 (0.061 ± 0.0061), an increase of approximately 64%. From 25% to 15% moisture content, the content decreased. The total polysaccharide content (Figure 2G) exhibited a similar trend. Sample X1 had the lowest polysaccharide content of 4.256 ± 0.832, while sample X4 had the highest content among the untreated samples at 6.534 ± 0.657, an increase of approximately 53%. In the kneaded and sweated samples, polysaccharide content increased with decreasing moisture content from 55% to 25%, peaking at 8.998% ± 0.872% in sample Y5, an increase of approximately 43% compared to Y1. The kneading and sweating treatment had a significant impact on the content of polysaccharides and calycosin-7-O-β-D-glucopyranoside. Compared to unpretreated samples, the total polysaccharides and calycosin-7-O-β-D-glucopyranoside contents in treatment samples Y1-Y6 were significantly higher at moisture levels of 15%, 25%, and 45%. At 55% moisture content, only the calycosin-7-O-β-D-glucopyranoside content was significantly higher, while at 35% moisture content, only the polysaccharide content increased significantly. The content of astragaloside IV showed no significant difference between the treated and untreated samples. This suggests that the kneading and sweating treatment was more effective in increasing the content of polysaccharides and calycosin-7-O-β-D-glucopyranoside.

#### 3.1.4 Entropy Weight-TOPSIS analysis

Entropy weight-TOPSIS analysis provides a more comprehensive evaluation than methods relying on a single aspect of quality. In this study, the quality of samples was influenced by a combination of appearance and intrinsic content. The entropy weight-TOPSIS analysis were performed based on appearance scores, total ash content, water-soluble extract content, total polysaccharides, astragaloside IV, and calycosin-7-O-β-D-glucopyranoside contents (Tables 2, 3). Samples Y5 and Y4 ranked first and second, respectively. Which can be attributed to their strong performance in appearance scores and extract content. The high levels of metabolites, especially total polysaccharides and astragaloside IV, significantly contributed to these results. In contrast, samples X5 and X4 ranked 6th and 8th, respectively, further confirming the efficacy of the kneading and sweating treatment methods. Therefore, cutting at a moisture content of 25%–35% after kneading and sweating treatment is identified as the optimal moisture level and processing method for AR.

**TABLE 2:** Entropy weight method for calculating weights.

Evaluation indicator	Information entropy (e)	Information utility (d)	Weight coefficient (w%)
Appearance score	0.915	0.085	14.704
Extractable content	0.872	0.128	22.133
Total polysaccharides	0.897	0.103	17.788
Astragaloside IV	0.860	0.140	24.139
Calycosin-7-O-β-D-glucopyranoside	0.945	0.055	9.440
Total ash	0.932	0.068	11.796

**TABLE 3:** TOPSIS evaluation results of different processing Methods for AR.

Sample	Distance to positive ideal solution (D^+^)	Distance to negative ideal solution (D^−^)	Relative closeness (C_*i*_)	Ranking
Y5	0.223	0.907	0.803	1
Y4	0.439	0.691	0.611	2
Y3	0.422	0.662	0.611	3
Y6	0.511	0.732	0.589	4
X6	0.509	0.629	0.553	5
X4	0.554	0.609	0.524	6
Y1	0.635	0.559	0.468	7
X5	0.624	0.515	0.452	8
X2	0.661	0.538	0.449	9
X3	0.608	0.479	0.441	10
Y2	0.646	0.491	0.432	11
X1	0.939	0.343	0.268	12

### 3.2 Comparison of appearance between AR-F and AR-T

**FIGURE 3:**
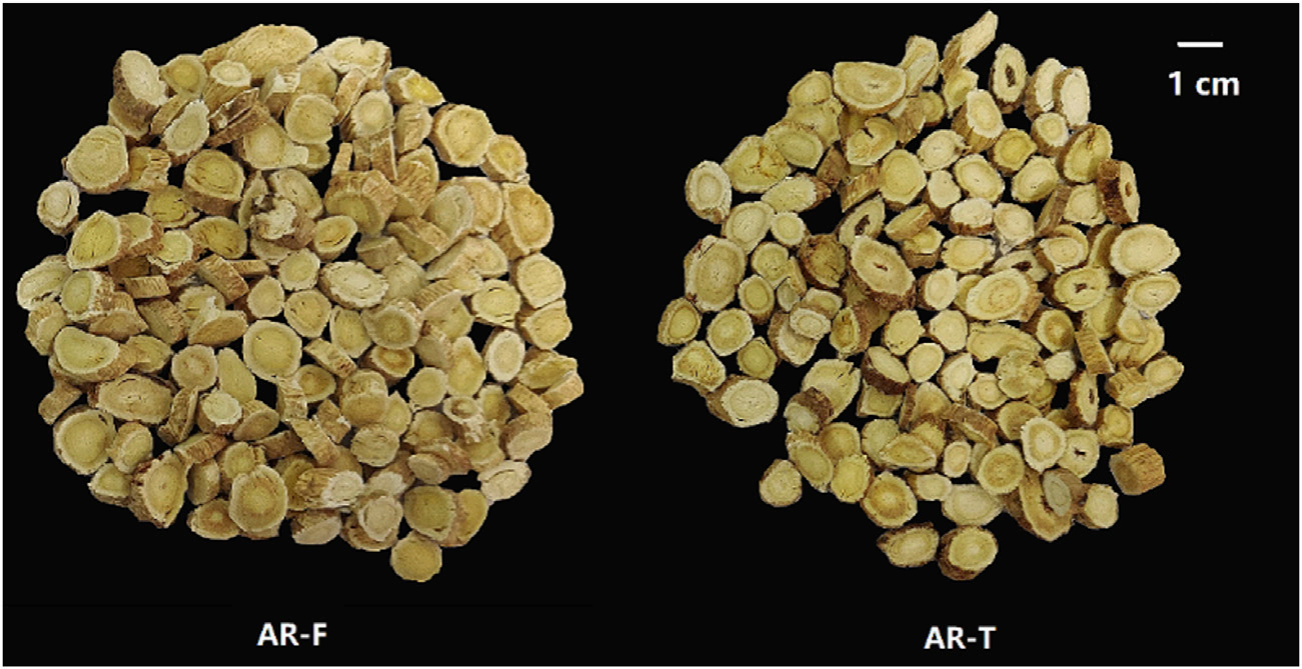
Appearance of AR-F and AR-T.

The appearances of AR-F and AR-T are shown in Figure 3. It can be seen that the morphologies of AR-F and AR-T are largely similar. Both are thick slices with orbicular or elliptic shapes, featuring visible longitudinal wrinkles or grooves. The cut surfaces are yellowish-white in the cortex and light yellow in the xylem, with radial textures and fissures. The main difference is the slightly lighter color of the outer skin when freshly cut, which is closer to the color of the root.

### 3.3 Comparison of active metabolites contents between AR-F and AR-T

#### 3.3.1 Contents of total polysaccharides, saponins, and flavonoids

Total polysaccharides, saponins and flavonoids are the three active metabolites responsible for the pharmacological effects of AR, including antioxidant, antiviral, antitumor activities, reduction of insulin resistance, protection against myocardial ischemia, and immune regulation (Cao et al., 2024). Their contents are shown in Table 4. The total polysaccharides and flavonoids contents in AR-F are significantly higher than those in AR-T, while the total saponins content shows no significant difference between them.

**TABLE 4:** Average content values and significance for AR-F and AR-T (n = 3, X±SD).

Content (mg/g)	AR-F	AR-T
Total Polysaccharides	85.152 ± 5.250^**^	55.640 ± 1.770
Total Saponins	14.348 ± 0.751	14.075 ± 1.099
Total Flavonoids	7.808 ± 0.015^**^	7.378 ± 0.129

Compared with AR-T, ^*^ *p* < 0.05,^**^ *p* < 0.01.

#### 3.3.2 Monosaccharide composition analysis

**FIGURE 4:**
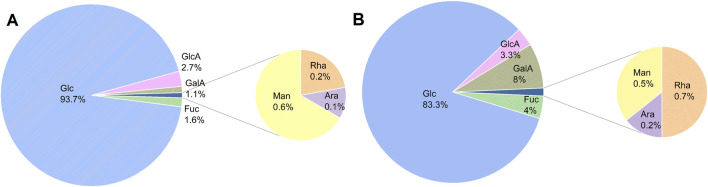
Monosaccharide composition analysis of AR. **(A)** AR-F. **(B)** AR-T.

The types of monosaccharides are closely related to the biological activity of polysaccharides. Monosaccharide composition analysis showed that both AR-F and AR-T polysaccharides consist of seven monosaccharides: glucose, galactose, fucose, rhamnose, arabinose, mannose, and galacturonic acid, with glucose being the major monosaccharide. This is consistent with previous reports (Li et al., 2023a; Li et al., 2023b; Guo et al., 2024), although the relative molar ratios of the monosaccharides differ, as shown in Figure 4. Moreover, literature indicates that polysaccharides with a high proportion of glucose exhibit stronger radical scavenging and anti-inflammatory activities *in vitro* (Liao et al., 2018).

#### 3.3.3 Saponin metabolite analysis

**FIGURE 5:**
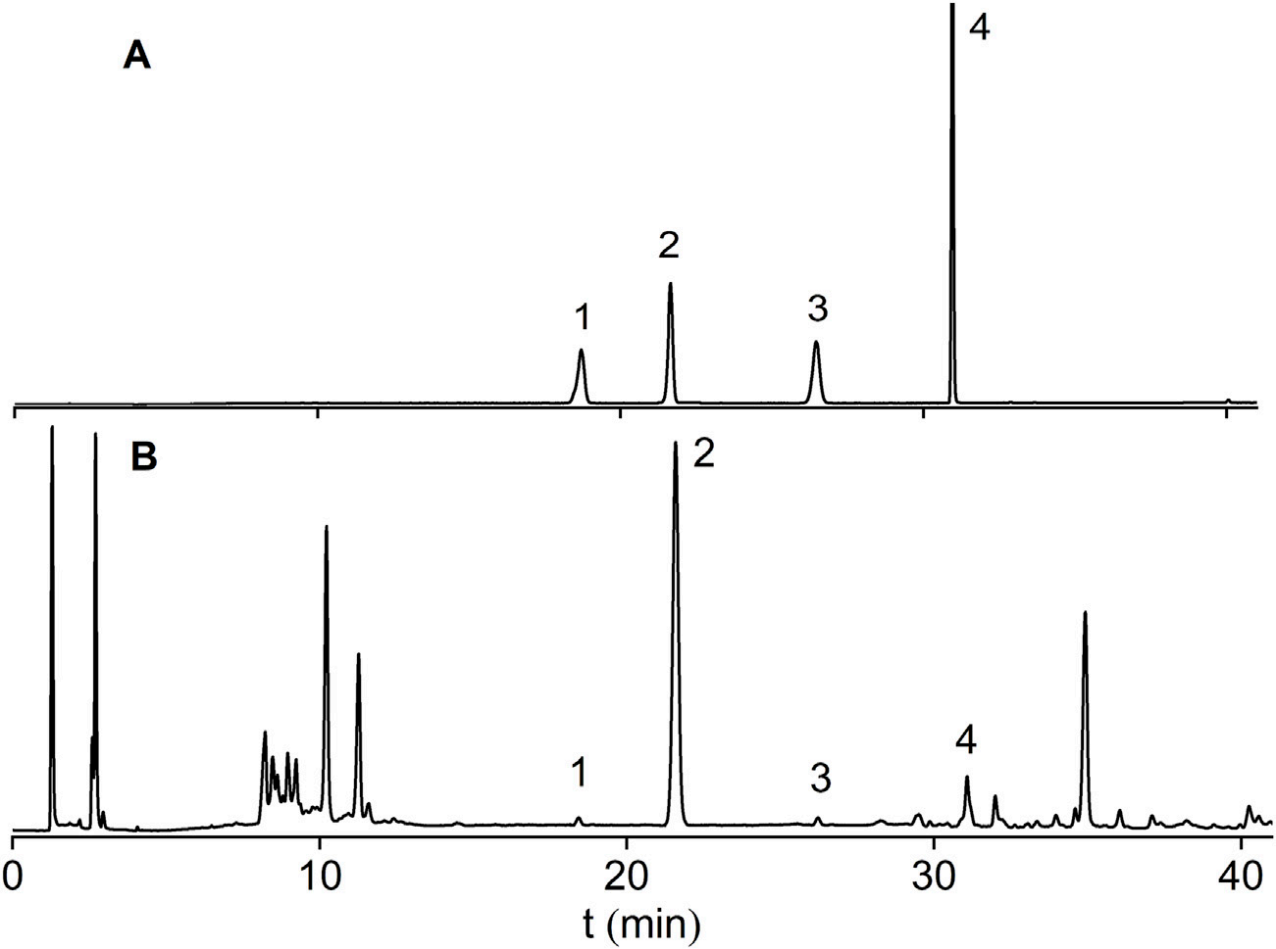
HPLC-ELSD chromatograms of saponin standards and AR. **(A)** Mixed Standards. **(B)** AR Samples; Peaks:1 Astragaloside III; 2 Astragaloside IV; 3 Astragaloside II; 4 Astragaloside I.

**FIGURE 6:**
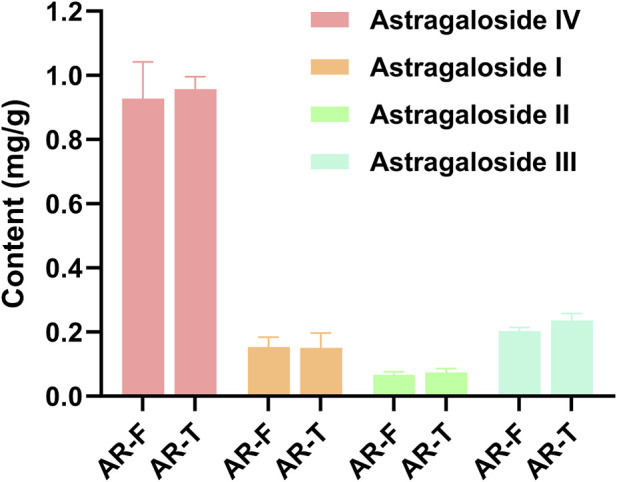
Content of four saponin metabolites in AR-F and AR-T Compared with AR-T, ^*^ *p* < 0.05, ^**^ *p* < 0.01.

Astragaloside I-IV are the major saponin metabolites in AR. The HPLC-ELSD chromatograms of mixed standard samples and AR samples are shown in Figure 5, and the regression equations for each metabolite are listed in Table 5. The content determination results are presented in Figure 6. No significant differences were observed in the content of each saponin between AR-F and AR-T, which is consistent with the total saponins content results.

**TABLE 5:** Linear regression equations for saponin standards in AR.

Metabolite	Linear regression equation	*R* ^2^	Linear range (μg/mL)
Astragaloside IV	In*Y* = 1.1785 In*X* – 0.1987	0.9999	8.3360 ∼ 1,042.0000
Astragaloside I	In*Y* = 1.4270 In*X* – 1.7119	0.9994	7.6160 ∼ 952.0000
Astragaloside II	In*Y* = 1.6145 In*X* – 2.6153	0.9995	7.3920 ∼ 824.0000
Astragaloside III	In*Y* = 1.0790 In*X* – 0.1688	0.9992	8.5920 ∼ 1,074.0000

#### 3.3.4 Flavonoid metabolite analysis

**FIGURE 7:**
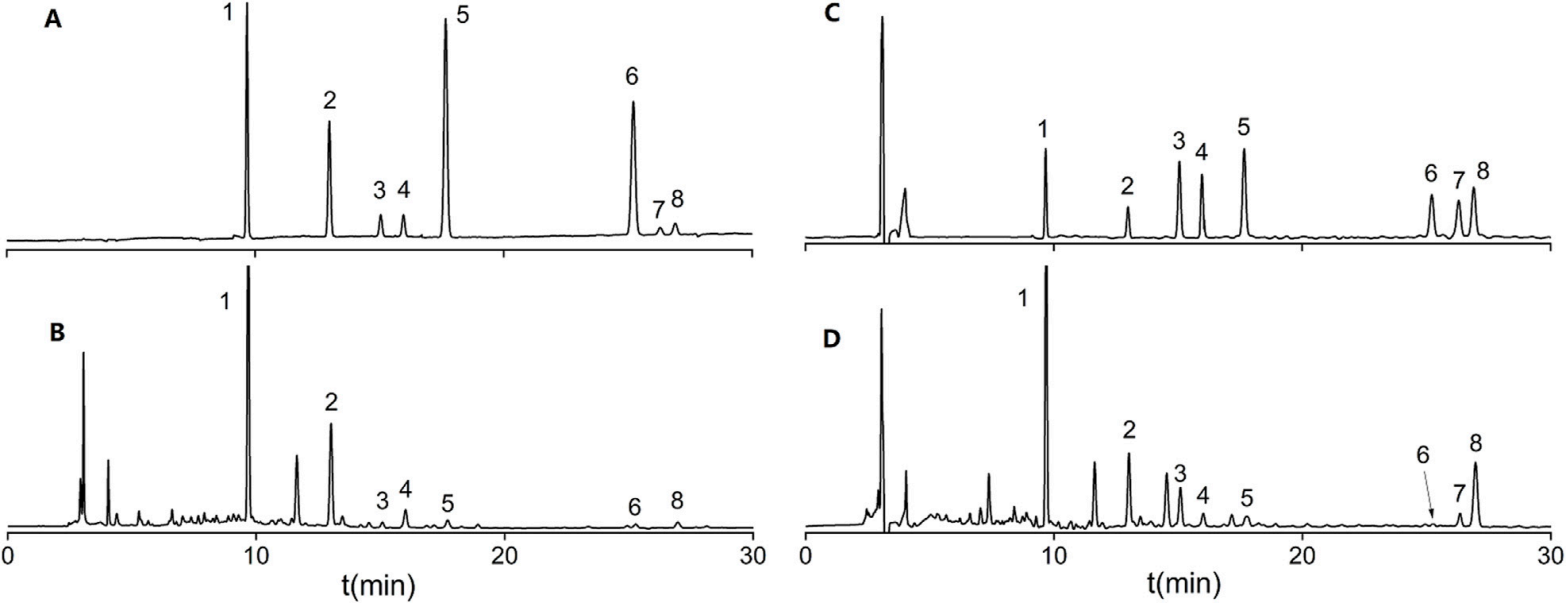
HPLC-DAD chromatograms of flavonoid standard mixtures and AR. **(A)** Mixed standard at 260 nm. **(B)** AR at 260 nm. **(C)** Mixed standard at 230 nm. **(D)** AR at 230 nm; Peaks:1 Calycosin-7-O-β-D-glucoside; 2 Formononetin-7-O-β-D-glucoside; 3 Astrapterocarpan-7-O-β-D-glucoside; 4 Isomucronulatol-7-O-β-D-glucoside; 5 Calycosin; 6 Formononetin; 7 Astrapterocarpan; 8 Isomucronulatol.

**FIGURE 8:**
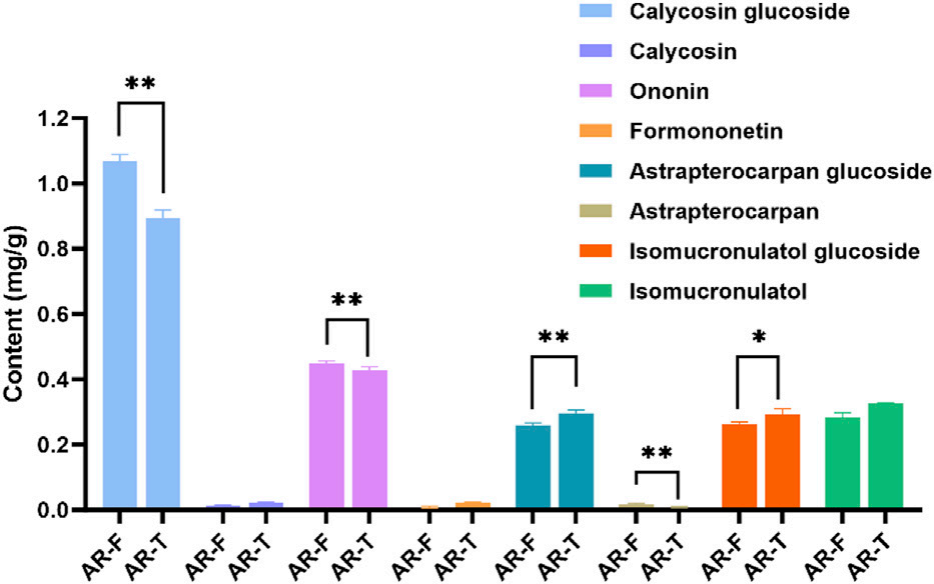
Content of eight flavonoid metabolites in AR-F and AR-T. Compared with AR-T, ^*^ *p* < 0.05, ^**^ *p* < 0.01.

Flavonoids are widely reported for their pharmacological activities (Cao et al., 2024). Notably, astrapterocarpan-7-O-β-D-glucoside, isomucronulatol-7-O-β-D-glucoside and isomucronulatol are unique metabolites of Astragalus (Guo et al., 2022; Lian et al., 2023). The HPLC-DAD chromatograms of the mixed standard samples and AR samples are shown in Figure 7, and the regression equations for each metabolite are listed in Table 6. The content determination results are shown in Figure 8. In AR-F, calycosin-7-O-β-D-glucoside, formononetin-7-O-β-D-glucoside, and astrapterocarpan are present at higher levels, while in AR-T, higher levels of calycosin, formononetin, astrapterocarpan-7-O-β-D-glucoside, isomucronulatol-7-O-β-D-glucoside, and isomucronulatol are observed. The cumulative content of these eight flavonoids is higher in AR-F compared to AR-T, which aligns with the total flavonoid content results. These flavonoids can be categorized into glycosides (calycosin-7-O-β-D-glucoside, formononetin-7-O-β-D-glucoside, astrapterocarpan-7-O-β-D-glucoside, and isomucronulatol-7-O-β-D-glucoside) and aglycones (calycosin, formononetin, astrapterocarpan, and isomucronulatol). The glycoside/aglycone ratio is a good indicator of AR’s growth, developmental status, and stress resistance (Chen et al., 2022b). In AR-F and AR-T, the glycoside/aglycone ratios are 6.33 and 5.04, respectively, indicating a higher trend of glycoside synthesis in AR-F compared to AR-T.

**TABLE 6:** Linear regression equations for flavonoid standards in AR.

Metabolite	Linear regression equation	*R* ^2^	Linear range (μg/mL)
Calycosin-7-O-β-D-glucoside	*Y* = 27.865*X* + 13.501	0.9998	20.040 ∼ 200.400
Calycosin	*Y* = 43.641*X* + 19.567	0.9997	1.080 ∼ 43.200
Formononetin-7-O-β-D-glucoside	*Y* = 24.533*X* - 2.993	0.9999	4.106 ∼ 60.584
Formononetin	*Y* = 36.367*X* + 7.222	0.9999	2.100 ∼ 42.000
Astrapterocarpan-7-O-β-D-glucoside	*Y* = 15.141*X* + 24.077	0.9995	12.250 ∼ 122.500
Astrapterocarpan	*Y* = 28.775*X* + 51.599	0.9999	8.800 ∼ 176.000
Isomucronulatol-7-O-β-D-glucoside	*Y* = 15.618*X* + 6.137	0.9998	10.400 ∼ 208.000
Isomucronulatol	*Y* = 32.854*X* + 28.166	0.9999	10.204 ∼ 102.040

### 3.4 Comparison of pharmacological mechanism between AR-F and AR-F

#### 3.4.1 Targets prediction of flavonoid metabolites

Fresh processing and traditional processing do not result in differences in the types of metabolites (Sun et al., 2024). However, preliminary studies revealed that the total flavonoid content in AR-F was significantly higher than that in AR-T. Based on this, flavonoid metabolites were selected as a focus to explore the mechanism underlying the differences in pharmacological effects. A total of 35 flavonoid metabolites were identified through preliminary studies, literature review, and screening through SwissADME database (Table 7). Additionally, 460 potential targets were predicted through SwissTargetPrediction database.

**TABLE 7:** Potential medicinal metabolites of flavonoids in AR.

No.	Metabolite	No.	Metabolite
1	Calycosin	19	7,3ʹ-dihydroxy-8,4ʹ-dimethoxyisoflavone
2	Calycosin-7-O-β-D-glucoside	20	3ʹ,7-Dihydroxy-5ʹ-methoxyisoflavone
3	Formononetin	21	4ʹ,5,7-Trihydroxy-3ʹ-methoxyisoflavone
4	Formononetin7-O-glucoside	22	Afrormosin
5	Astrapterocarpan	23	3,9,10-Tri-methoxypterocarpan
6	Astrapterocarpan-7-O-β-D-glucoside	24	Daidzein
7	Isomucronulatol	25	8,2ʹ-Dihydroxy-7,4ʹ-dimethoxyisoflavane
8	4ʹ,7-dihydroxyflavone	26	7-O-methylisomucronulatol
9	3ʹ,4ʹ,7-trihydroxyflavone	27	7,2ʹ,3ʹ-Trihydroxy-4ʹ-methoxy-isoflavane
10	Oroxylin-A	28	Pendulone
11	Wogonin	29	LicochalconeB
12	Liquiritigenin	30	isoliquiritigenin
13	Quercetin	31	2ʹ-Methoxyisoliquiritigenin
14	Kaempferol	32	4,4ʹ,6ʹ-Trihydroxychalcone
15	Genistein	33	4-Methoxy-4ʹ,6ʹ-dihydroxychalcone
16	Pratensein	34	4,4ʹ-Dimethyl-6ʹ-hydroxychalcone
17	3ʹ,7,8-trihydroxy-4ʹ-methoxyisoflavone	35	Sophorophenolone
18	8,3ʹ-dihydroxy-7,4ʹ-dimethoxyisoflavone		

#### 3.4.2 Construction of the PPI network and identifcation of core targets

**FIGURE 9:**
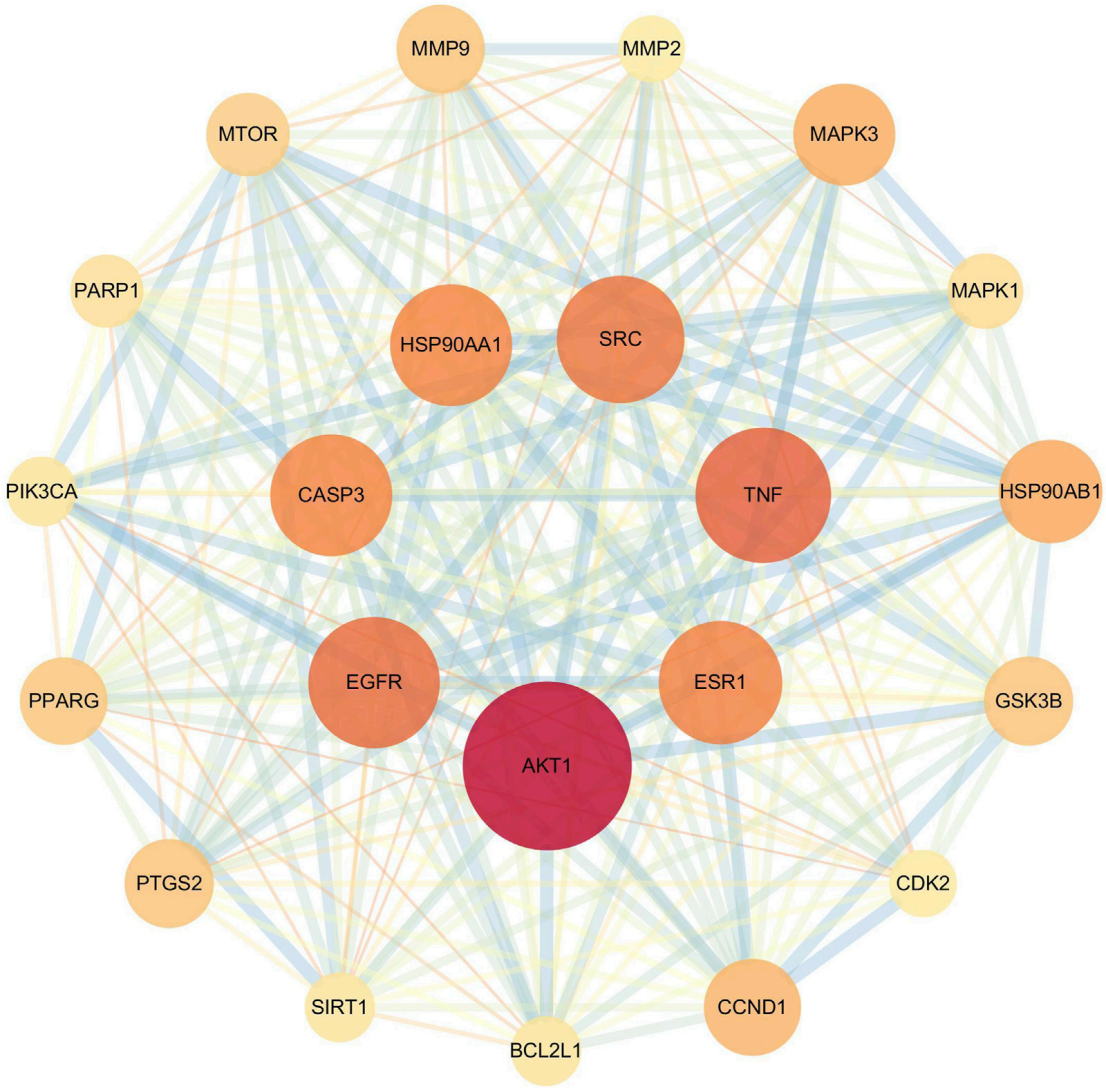
PPI network of core targets of flavonoid metabolites in AR.

PPI network analysis and core target screening of the 460 potential targets revealed that the core targets of flavonoid metabolites were primarily AKT1, TNF, EGFR, SRC, ESR1, HSP90AA1, and CASP3 (Figure 9).

#### 3.4.3 Enrichment analyses using GO and reactome databases

**FIGURE 10:**
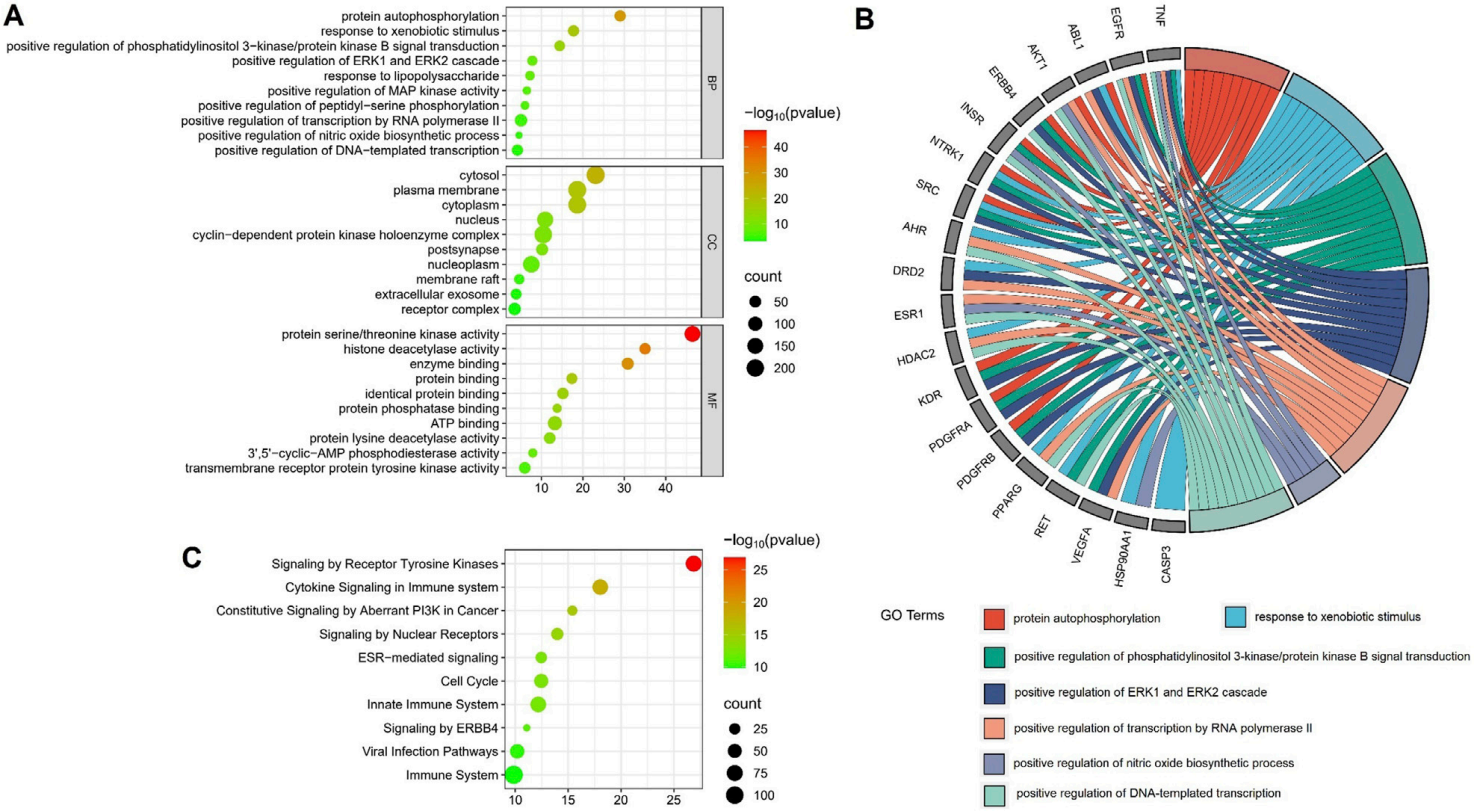
GO **(A, B)** and REACTOME Pathway **(C)** enrichment analysis.

The potential targets of the flavonoid metabolites were analyzed through GO enrichment, which identified 136 biological processes, 77 cellular components, and 150 molecular functions. The main biological processes included protein autophosphorylation, response to xenobiotic stimulus, and positive regulation of phosphatidylinositol 3-kinase/protein kinase B signal transduction. The main cellular components included the cytosol, plasma membrane, and cytoplasm, while the primary molecular functions included protein serine/threonine kinase activity, histone deacetylase activity, and enzyme binding (Figure 10A). These findings indicated that the main biological processes involved were protein autophosphorylation and response to xenobiotic stimulus. GO chord maps revealed that the targets AKT1, EGFR, TNF, ESR1, and SRC were enriched in these processes (Figure 10B). REACTOME pathway enrichment analysis identified a total of 304 signaling pathways enriched for the 460 potential targets of flavonoid metabolites. Clustering analysis showed that these pathways were primarily involved in signal transduction and immune system functions. The bubble diagrams indicated that key pathways within these categories included signaling by receptor tyrosine kinases, cytokine signaling in the immune system, and constitutive signaling by aberrant PI3K in cancer (Figure 10C).

#### 3.4.4 Molecular docking analysis

**FIGURE 11:**
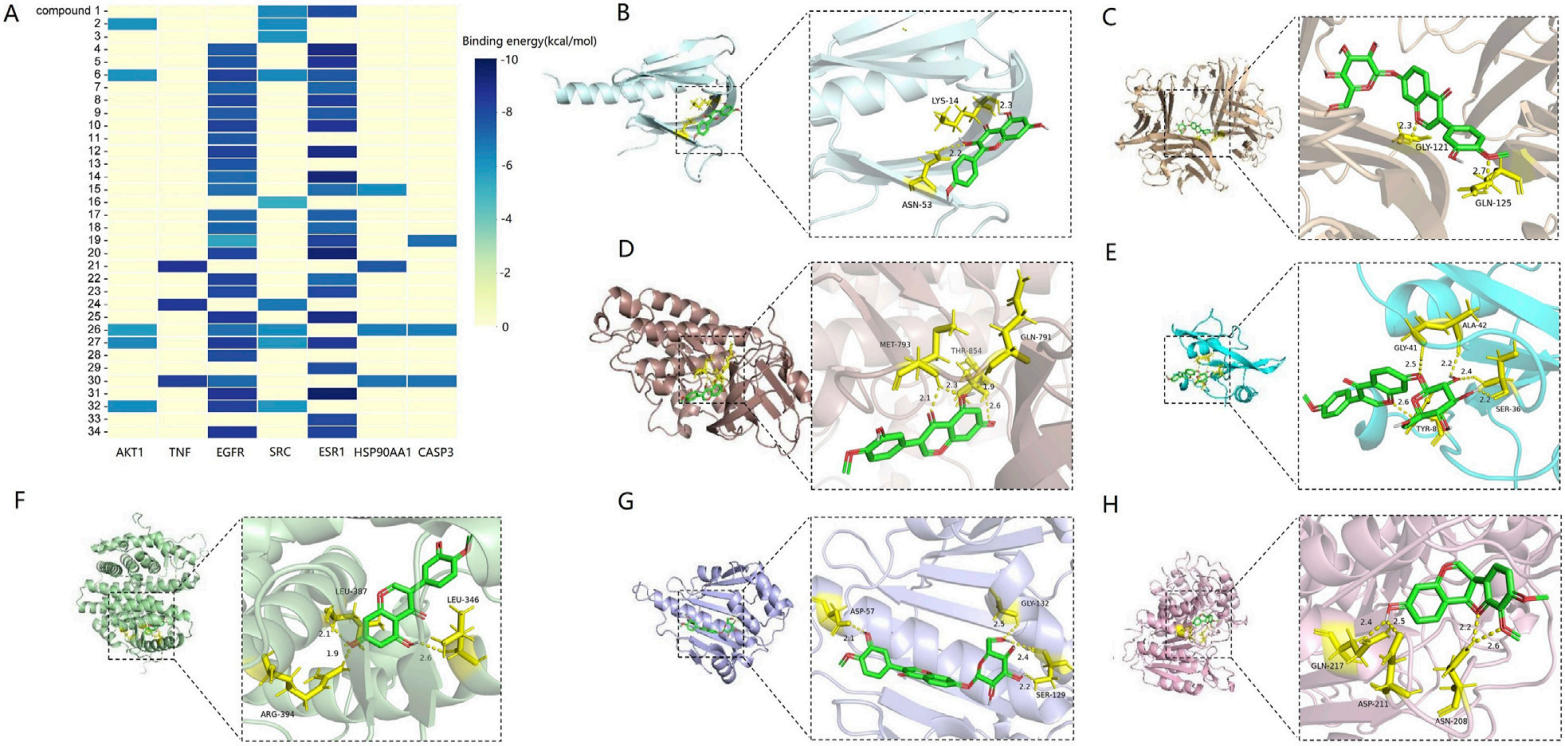
Molecular docking results **(A)** heatmap of binding energy between flavonoids and core targets. **(B)** 3D binding conformation of AKT1 and kaempferol. **(C)** 3D binding conformation of TNF and calycosin-7-O-β-D-glucoside. **(D)** 3D binding conformation of EGFR and pratensein. **(E)** 3D binding conformation of SRC and formononetin-7-O-β-D-glucoside. **(F)** 3D binding conformation of ESR1 and pratensein. **(G)** 3D binding conformation of HSP90AA1 and calycosin-7-O-β-D-glucoside. **(H)** 3D binding conformation of CASP3 and astrapterocarpan.

The molecular docking results revealed that the binding energies between the seven core targets and the flavonoid metabolites were all greater than 5 kcal/mol, with Pratensein showing the highest binding energy to SRC (−9.6 kcal/mol). These findings suggest that the flavonoid metabolites bind well to the core targets (Figure 11A). Additionally, the highest scoring results for each target were visualized (Figures 11B–H).

#### 3.4.5 Immunomodulatory effects

**FIGURE 12:**
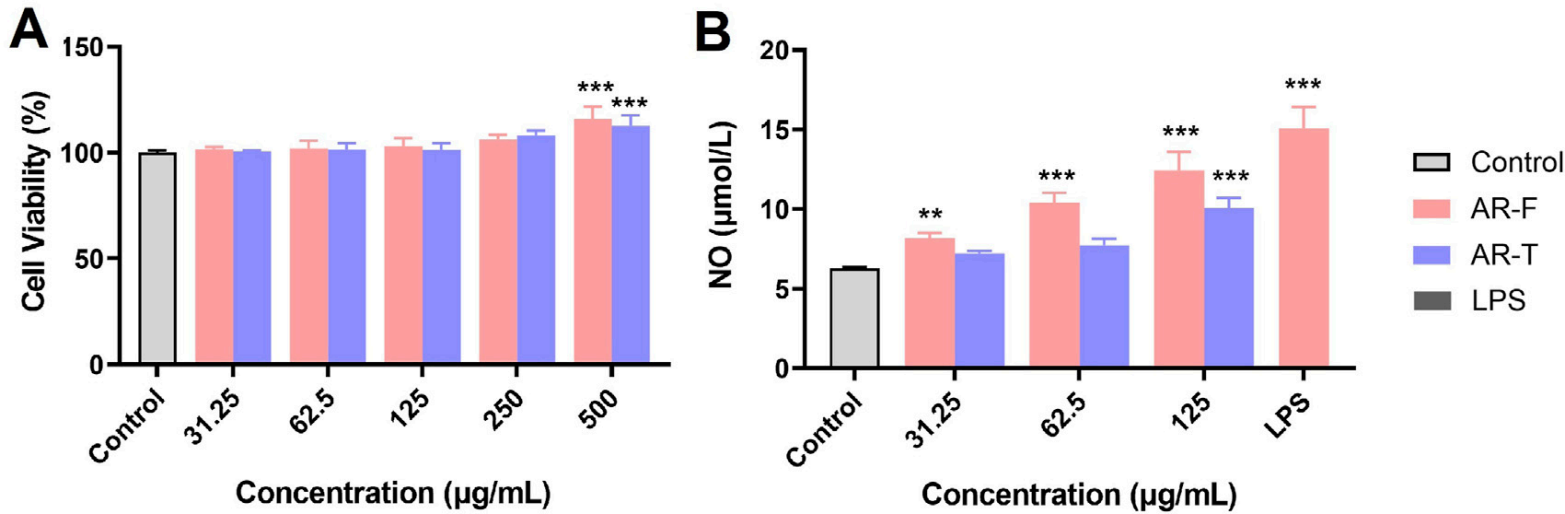
Immunomodulatory activity measurements of TFA in AR-F and AR-T. **(A)** RAW264.7 cell viability. **(B)** NO secretion by RAW264.7 cells. Compared with control, ^**^ *p* < 0.01.

The impact of different concentrations of TFA on RAW264.7 cell viability is shown in Figure 12A. Within the concentration range of 31.25–125 μg/mL, neither AR-F nor AR-T significantly affects cell viability. However, at concentrations of 500–1,000 μg/mL, both AR-F and AR-T promote cell proliferation. The effect of TFA on NO secretion of cells is shown in Figure 12B. At concentrations of 62.5 and 125 μg/mL, both TFA in AR-F and AR-T significantly promote NO secretion compared to the control group, with AR-F showing higher NO levels than AR-T at the same concentrations. These results suggest that AR-F has better immunomodulatory activity compared to AR-T.

#### 3.4.6 Enzyme-linked immunosorbent assay

**FIGURE 13:**
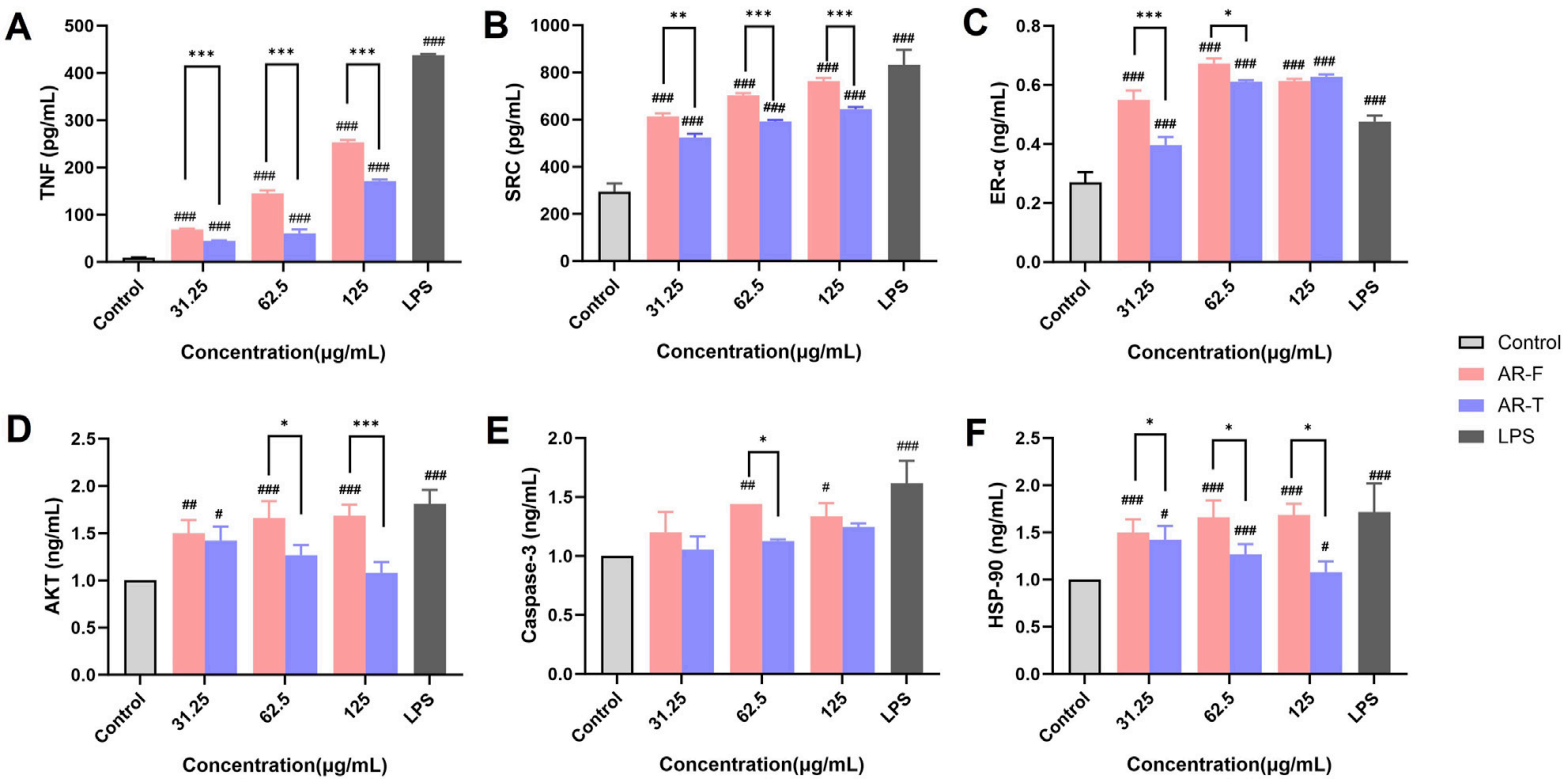
Enzyme-linked immunosorbent (ELISA) assay. **(A)** Tumor necrosis factor (TNF) **(B)** Proto-oncogene tyrosine-protein kinase Src (SRC) **(C)** Estrogen receptor (ER-α) **(D)** Protein kinase B (AKT) **(E)** Caspase-3 **(F)** Heat shock protein HSP 90-α (HSP-90) Compared with control, ^#^ *p* < 0.05, ^##^ *p* < 0.01, ^###^ *p* < 0.001; compared with AR-T, ^*^ *p* < 0.05, ^**^ *p* < 0.01, ^***^ *p* < 0.001.

The effect of TFA in AR-F and AR-T on the secretion of core target proteins in RAW264.7 cell is presented in Figure 13. Both AR-F and AR-T significantly increased the protein contents of TNF, SRC, ER-α, AKT, Caspase-3 and HSP-90 compared to the control group, with AR-F exhibiting higher levels than AR-T.

Specifically, compared to the AR-T group, TNF, SRC and HSP-90 content in the AR-F group was significantly elevated within the concentration range of 31.25–125 μg/mL (Figures 13A, B, F). Similarly, ER-α and AKT contents in the AR-F group were significantly increased in the concentration range of 31.25–62.5 μg/mL and 62.5∼125 μg/mL specifically (Figures 13C–D) compared to the AR-T group. For Caspase-3, a significant increase was observed in the AR-F group at concentrations of 62.5 μg/mL (Figure 13E) compared to the AR-T group.

## 4 Discussion

The color, shape, and content of active metabolites in medicinal slices are crucial indicators of the quality of TCM. Our study revealed that moisture content, as well as kneading and sweating treatment, significantly affect these indicators during fresh processing. Specifically, slicing fresh medicinal materials with high moisture content results in a whiter cross-section, while materials with lower moisture content yield a yellower cross-section. This difference may be related to the fact that high moisture content slows down the oxidation of polyphenolic metabolites and enzymatic browning in AR (Bai et al., 2020). Furthermore, as moisture content decreases, the levels of polysaccharides and calycosin-7-O-glucoside in Astragalus initially increase and then decrease. This could be due to high moisture content in fresh materials maintaining enzyme activity and cellular metabolism, which continues to participate in biosynthesis and increase the content of polysaccharides and flavonoids. However, as the AR continues to dry, enzyme activity gradually diminishes, halting the biosynthesis process. Prolonged drying may also lead to oxidation and degradation of effective metabolites, causing a decrease in their content (Bai et al., 2020). Kneading and sweating treatments can improve the morphology of AR-F. This may be because these processes make the fibrous tissue of the material more pliable, facilitating the connection between the skin and wood, and reducing the separation of the skin and flesh in slices. Additionally, sweating can redistribute the moisture within the semi-dried AR, reducing the moisture difference between the interior and the surface, and thereby decreasing the rate of fragment formation. Furthermore, the significantly increased contents of polysaccharides and calycosin-7-O-glucoside observed in the kneading and sweating treatment group compared to the unpretreated group may be due to the disruption the cell wall structure by these processes, which accelerates enzymatic reactions and promotes the release and transformation of polysaccharides and flavonoids (Dai et al., 2018). Comparing the content of various metabolites between AR-F and AR-T, we found that total polysaccharides and total flavonoids in AR-F were significantly higher than those in AR-T, consistent with findings for fresh processing of *Ophiopogon japonicus* (Cai et al., 2023). This could be attributed to the fact that fresh materials, compared to traditional drying processes, undergo less thermal degradation, oxidation, and loss of water-soluble metabolites. Notably, the content of calycosin-7-O-glucoside, a key quality control metabolite, was significantly higher in AR-F, indicating that the fresh processing method is an important way to improve the quality of AR. These findings provide valuable guidance for production and processing of AR in the pharmaceutical industry.

AR contains polysaccharides, flavonoids, and saponins as its major bioactive compounds. We conducted a comparative analysis of the chemical profiles of AR-F and AR-T. The results showed a significant increase in polysaccharides and flavonoids in AR-F compared to AR-T. Notably, the contents of calycosin-7-O-β-D-glucoside, ononin, and astrapterocarpan in AR-F increased by approximately 19.5%, 5.4%, and 77.8%, respectively.

Calycosin-7-O-β-D-glucoside, formononetin-7-O-β-D-glucoside, and astrapterocarpan are natural compounds predominantly found in legumes, especially in AR. Studies have demonstrated that calycosin-7-O-β-D-glucoside and formononetin-7-O-β-D-glucoside exhibit bidirectional immunomodulatory effects, benefiting inflammatory conditions. Calycosin-7-O-β-D-glucoside enhances macrophage phagocytosis and immune activity, promotes T and B cell proliferation, and strengthens host defense against pathogens. Additionally, it suppresses the excessive release of inflammatory cytokines such as TNF-α, IL-1β, and IL-6, alleviating inflammation caused by hyperactive immune responses. This compound also modulates the Th1/Th2 and Treg/Th17 balance, maintaining immune tolerance and controlling chronic inflammation and autoimmune diseases (Gao et al., 2014).

Formononetin-7-O-β-D-glucoside promotes B cell proliferation and antibody production, regulates cytokine secretion by T cells, and balances Th1/Th2 responses. It inhibits NF-κB and MAPK signaling pathways, reducing pro-inflammatory cytokine production and mitigating inflammation. Furthermore, it supports intestinal barrier integrity and modulates gut microbiota, indirectly enhancing immune functions (Che et al., 2024). As an antioxidant, it reduces reactive oxygen species (ROS)-induced damage to immune cells and increases anti-inflammatory cytokine expression. Astrapterocarpan interferes with NLRP3 inflammasome activation, suppressing the release of pro-inflammatory cytokines like IL-1β and IL-18, thereby attenuating inflammation. Research suggests that it may regulate the Treg/Th17 balance, modulating immune tolerance and inflammation. Additionally, it decreases oxidative stress markers (e.g., malondialdehyde, MDA) and enhances antioxidant enzyme activity, protecting immune cells from damage.

Existing studies indicate that the fresh-cutting and traditional drying processes do not alter the chemical composition of AR but affect the relative contents of specific compounds. To elucidate the pharmacological differences between AR-F and AR-T, we conducted a network pharmacology analysis focusing on total flavonoids, which differ significantly in content between the two. PPI network analysis identified seven core targets for total flavonoids in AR (TFA): AKT1, ESR1, TNF, SRC, HSP90AA1, CASP3, and EGFR. These core targets are closely related to cytokine signaling and receptor tyrosine kinase pathways in the immune system. Among these targets, AKT1 is a central node influencing immune responses by regulating T cell activation and proliferation. It also participates in modulating inflammation and phagocytosis in macrophages and dendritic cells. ESR1, TNF, SRC, HSP90AA1, and CASP3 further contribute to immunoregulation via distinct mechanisms: ESR1 influences the activity of T and B cells and plays a role in the treatment of sex-related autoimmune diseases, such as rheumatoid arthritis and systemic lupus erythematosus. TNF regulates inflammatory responses, including cytokine secretion, leukocyte recruitment, and angiogenesis. Its overexpression is associated with autoimmune diseases such as Crohn’s disease and psoriasis. SRC, a tyrosine kinase, is involved in signal transduction, cell migration, and proliferation. It regulates T and B cell receptor signaling and functions in natural killer cells and dendritic cells. HSP90AA1 modulates inflammatory responses by affecting type I interferon and NF-κB signaling pathways and serves as a chaperone for key molecules in antigen presentation. CASP3 regulates immune cell apoptosis and maintains immune homeostasis. In autoimmune diseases, aberrant CASP3 activity may lead to insufficient or excessive apoptosis.

To validate the effects of AR-F and AR-T total flavonoids on these core targets, we performed cell-based experiments using RAW264.7 macrophages. At concentrations of 31.25–125 μg/mL, AR-T significantly increased TNF secretion compared to the control group, consistent with literature reports (Guo et al., 2016). Notably, AR-F further enhanced TNF secretion compared to AR-T. Molecular docking results revealed that calycosin-7-O-β-D-glucoside exhibited the highest binding energy with TNF and HSP, ononin with SRC, and astrapterocarpan with CASP3. These findings suggest that the enhanced immunomodulatory activity of AR-F relative to AR-T is associated with the increased content of these three compounds, acting through targets such as TNF, HSP, SRC, and CASP3.

## 5 Conclusion

Fresh processing of medicinal materials offers certain advantages, including better retention of effective metabolites, reduced storage and processing steps, lower production costs, and improved processing efficiency. Our study optimized the fresh processing method of AR, and compared the differences in active metabolite content and pharmacological effects between fresh cutting and traditional methods. It was found that fresh cutting has advantages in retaining polysaccharide and flavonoid contents, and demonstrates better immune modulation activities. This study provides scientific evidence for the quality control and rational application of fresh processing AR.

## Data Availability

The original contributions presented in the study are included in the article/supplementary material, further inquiries can be directed to the corresponding author.
